# miR-218 Involvement in Cardiomyocyte Hypertrophy Is Likely through Targeting REST

**DOI:** 10.3390/ijms17060848

**Published:** 2016-05-31

**Authors:** Jing-Jing Liu, Cui-Mei Zhao, Zhi-Gang Li, Yu-Mei Wang, Wei Miao, Xiu-Juan Wu, Wen-Jing Wang, Chang Liu, Duo Wang, Kang Wang, Li Li, Lu-Ying Peng

**Affiliations:** 1 Key Laboratory of Arrhythmias, Ministry of Education, Tongji University School of Medicine, Shanghai 200092, China; ljjdaisywin@163.com (J.-J.L.); zhaocuimei2000@126.com (C.-M.Z.); happylag5@163.com (Z.-G.L.); 14wang_yumei@tongji.edu.cn (Y.-M.W.); wikimiao@126.com (W.M.); wuxiujuan1992@163.com (X.-J.W.); 2015vivian_wang@tongji.edu.cn (W.-J.W.); linuxonly@163.com (C.L.); wangduotc@126.com (D.W.); tongji_wk@163.com (K.W.); 2 Research Center for Translational Medicine, Shanghai East Hospital, Tongji University School of Medicine, Shanghai 200120, China; 3 Department of Pathology and Pathophysiology, Tongji University School of Medicine, Shanghai 200092, China

**Keywords:** miR-218, cardiomyocyte, hypertrophy, REST

## Abstract

MicroRNAs (miRNAs) have been identified as key players in cardiomyocyte hypertrophy, which is associated with significant risks of heart failure. However, many microRNAs are still not recognized for their functions in pathophysiological processes. In this study, we evaluated effects of miR-218 in cardiomyocyte hypertrophy using both *in vitro* and *in vivo* models. We found that miR-218 was evidently downregulated in a transverse aortic constriction (TAC) mouse model. Overexpression of miR-218 is sufficient to reduce hypertrophy, whereas the suppression of miR-218 aggravates hypertrophy in primary cardiomyocytes induced by isoprenaline (ISO). In addition, we identified RE1-silencing transcription factor (REST) as a novel target of miR-218; it negatively regulated the expression of REST in hypertrophic cardiomyocytes and the TAC model. These results showed that miR-218 plays a crucial role in cardiomyocyte hypertrophy, likely via targeting REST, suggesting a potential candidate target for interfering hypertrophy.

## 1. Introduction

Cardiac hypertrophy is an important compensatory mechanism along with the continuous increase of cardiac afterload, which effectively improves heart contractility and maintains normal cardiac output after the impairments of cardiac function for a certain period of time. However, this kind of compensatory mechanism has limited effectiveness, which eventually resulted in decompensation of cardiac function and heart failure under sustained stress due to maladaptive changes [1,2]. In recent years, a lot of miRNAs have been shown to contribute to the control of cardiovascular disease, but the underlying molecular mechanisms in which how miRNAs regulate cardiac hypertrophy are still poorly understood.

MiRNAs are small, highly conserved noncoding RNAs which can perform significant functions in many regulatory networks by negatively controlling the expression of target genes. [3]. MiRNAs have also been shown to play a variety of roles in the development of cardiac hypertrophy [4,5,6]. Recent research showed that mice enduring pressure overload decreased the expression levels of cardiac miR-133; consistently, inhibition of miR-133 with an anti-miR-133 oligonucleotide induced cardiac hypertrophy *in vivo* [7,8]. miR-26 proved to inhibit the expression of GATA4, so that the decline of miR-26 levels could aggravate hypertrophy [9]. Moreover, as a cardiac specific microRNA, overexpression of miR-208 generated cardiac hypertrophy in mice by negatively controlling sex-determining region Y-box 6 protein [10]. miR-22 was found as a critical regulator in cardiomyocyte hypertrophy and cardiac remodeling in mice models [11]. miR-23a was also involved in the hypertrophic signals through regulating Foxo3a [12]. Indeed, many miRNAs could modulate the pathological process by inhibiting target genes that mediate related signaling pathways to trigger cardiac hypertrophy [13]. 

miR-218 is transcribed from an intron of *Slit2* and *Slit3* genes, and shares a high level of sequence conservation from humans and mice to zebrafish (*danio rerio*) and *xenopus lavis* acrossspecies [14]. Thus far, miR-218 has been demonstrated as a repressor to inhibit tumor cell invasion, migration, and proliferation by regulating multiple cancer phenotype-associated genes that play important roles in mTOR and *Wnt*-mediated signaling pathways [15,16]. Recent studies have shown that miR-218 is evidently downregulated in both hypertrophy mice models and the end-stage heart failure patients by microarray analysis [17,18], but the molecular mechanism of hypertrophic involvement by miR-218 remains unknown. 

In this study, we observed that miR-218 could attenuate hypertrophy of cardiomyocytes induced with ISO. We also identified *REST* as a novel target gene that was negatively regulated by miR-218 in hypertrophy process. In addition, the level of miR-218 was dynamically downregulated in hearts of a TAC model. These results provide evidence that miR-218 may involve ISO-induced cardiomyocyte hypertrophy through multiple pathways mediated by REST.

## 2. Results

### 2.1. Overexpression of miR-218 Attenuates ISO-Induced Hypertrophy in Cardiomyocytes

**Figure 1:**
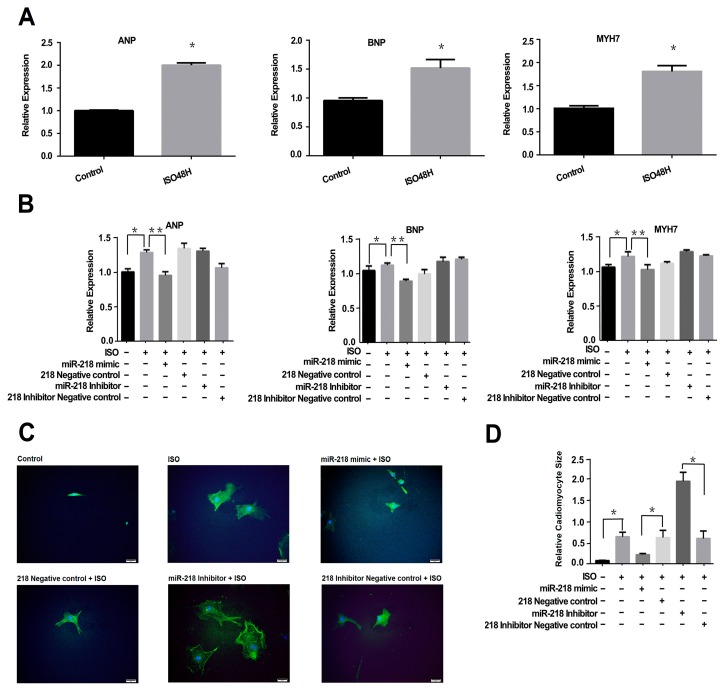
Effect of miR-218 on ISO-induced hypertrophy in cultured NRCMs. (**A**) the status of *ANP*, *BNP* and *MYH7* levels was detected by real-time PCR in induced-hypertrophic cadiomyocytes; (**B**) the effect of miR-218 mimic on expression status of *ANP*, *BNP* and *MYH7* in induced-hypertrophic cadiomyocytes; (**C**) the cell size change of cardiomyocytes in different conditions (Scale bar: 50 μm); and (**D**) mean cell size of α-actinin positive NRCMs. Fifty individual cells were analyzed per group. The data were displayed as the means ± SEM (* *p* < 0.05; ** *p* < 0.01).

To observe *in vitro* the change of miR-218 in hypertrophy, we induced Neonatal Rat Cardiac Myocytes (NRCMs) with 10 µM ISO for 48 h and checked the status of cardiac hypertrophy markers by real-time PCR. Indeed, the relative expression levels of Atrial Natriuretic Peptide (*ANP*), Brain Natriuretic Peptide (*BNP*) and b-Myosin Heavy Chain (*MYH7*) were all upregulated compared with those in control groups (Figure 1A). Under these conditions, the expression level of miR-218 was observed using a strategy of gain-of-function or loss-of-function in cardiomyocytes. We transfected NRCMs with synthetic chemicals including miR-218 mimic, miR-218 negative control, miR-218 inhibitor and miR-218 inhibitor negative control, respectively, and cultured the transfected cells with or without ISO for another 48 hours in order to induce hypertrophy. Overexpression of miR-218 by transfecting the mimic into NRCMs can significantly reduce the expression levels of hypertrophy-related genes (such as *ANP*, *BNP* and *MYH7*) (Figure 1B). In the meantime, the surface area of cardiomyocytes with overexpression of miR-218 was obviously decreased compared with control cells under the stimulation of ISO (Figure 1C,D). On the contrary, inhibiting the expression level of miR-218 can increase the surface area of cardiomyocytes induced by ISO (Figure 1C,D). These results indicate that miR-218 can attenuate cardiomyocyte hypertrophy *in vitro* by overexpression, suggesting that the small molecule plays a role in protecting cardiomyocyte hypertrophy from stimulation of ISO. 

### 2.2. miR-218 Expression Is Downregulated in TAC Model’s Heart

**Figure 2:**
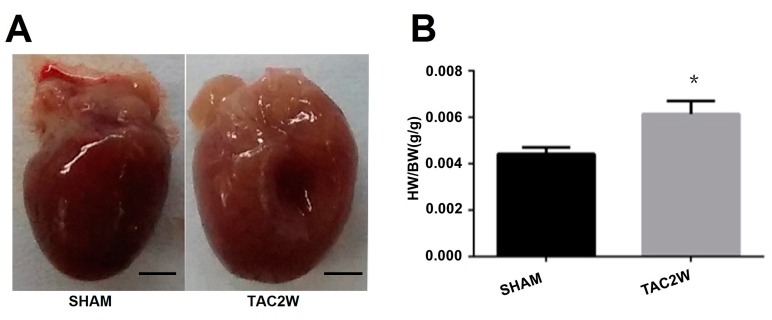

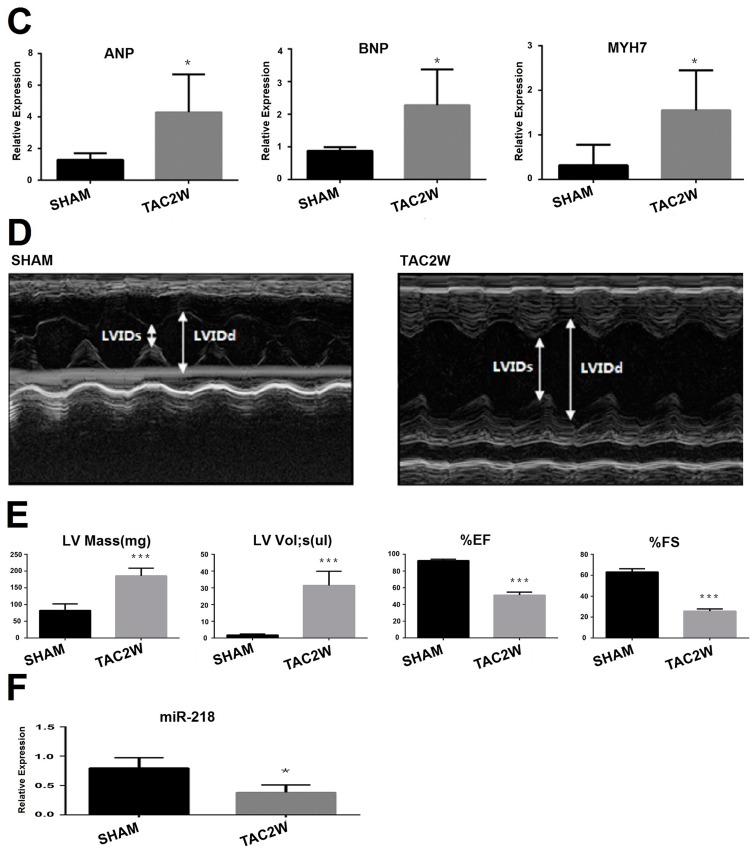
Downregulation of miR-218 in the heart of the two-week TAC (TAC2W) mouse model. (**A**) the change of gross morphology for the heart of the TAC model (Scale bar: 1 mm); (**B**) the change of weight-to-body ratio for the hearts of TAC-models (*n* = 13) and sham mice (*n* = 5); (**C**) the relative mRNA levels of *ANP*, *BNP* and *MYH7* in the hearts of TAC models (*n* = 13) and sham mice (*n* = 5); (**D**,**E**) the echocardiography results in two-week TAC models and the sham group (*n* = 3); and (**F**) the relative levels of miR-218 in two-week TAC-models (* *p* < 0.05; *** *p* < 0.001).

To detect the status of miR-218 expression with hypertrophy *in vivo*, we constructed a TAC mouse model based on a traditional strategy. Mice were randomly divided into two groups: a sham group and a TAC group. Two weeks after an overload stimulation, we found the heart size of mice in the TAC group to be significantly larger and the ratio of heart weight to body weight (HM/BM) was also dramatically increased, compared with that in the sham group, respectively (Figure 2A,B). The mRNA levels of *ANP*, *BNP* and *MYH7* were significantly upregulated in TAC model hearts (Figure 2C), and the echocardiography assessment also showed typical parameters of hypertrophy in the two-week TAC model. (Figure 2D,E). Meanwhile, miR-218 expression was also significantly downregulated in the mice with the pressure overload (Figure 2F), suggesting that miR-218 is indeed involved in hypertrophic process *in vivo*. 

### 2.3. miR-218 Regulates REST by Targeting the 3’-UTR of the Gene

**Figure 3:**
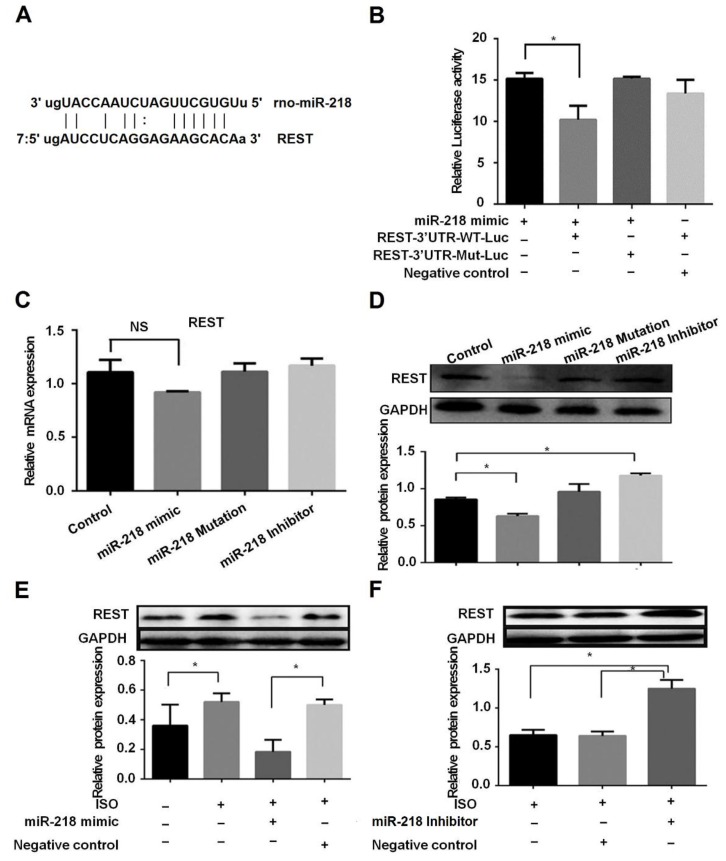

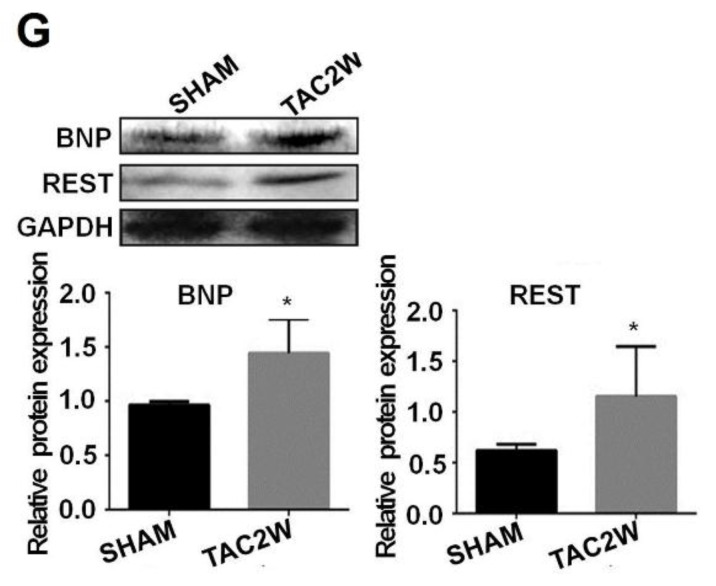
miR-218 targets the 3’-UTR of *REST*. (**A**) alignment of rat miR-218 sequence with the 3’-UTR of *REST*; (**B**) luciferase assay showed that miR-218 targets the 3’-UTR of *REST* in H9C2 cells; (**C**) the mRNA levels of *REST* in H9C2 cells transfected by miR-218 mimic; NS: not significantly different; (**D**) the protein expression level of REST in H9C2 cells under different conditions; (**E**) the regulation of miR-218 on the protein level of REST in NRCMs stimulated by ISO; (**F**) the regulation of miR-218 inhibitor on the protein level of REST in NRCMs stimulated by ISO; and (**G**) the expression levels of BNP and REST in two-week TAC models (*n* = 13) and sham mice (*n* = 5). GAPDH was used as internal control (* *p* < 0.05).

Given that miR-218 expression is downregulated in ISO-induced hypertrophic cardiomyocytes and TAC models, we next asked whether miR-218 regulates target genes related to the hypertrophic pathogenesis. REST has been shown to have a crucial role in the development of the heart [19]. Using bioinformatic approaches (miRanda) [20], we predicted *REST* as a potential target gene in which the 3’-untranslated region (3’-UTR) of the RNA transcript harbors the “seed” sequence of miR-218 (Figure 3A). To prove whether *REST* is targeted by miR-218, we use the *REST* 3’-UTR to replace the 3’-UTR of luciferase gene in a recombined pMIR-*REST* vector that carries a constitutively activated promoter for luciferase expression. We co-transfected the report plasmid into H9C2 cells with miR-218 mimic or control, and found that luciferase activity showed an obvious decrease in miR-218 mimics compared with that in control cells. In a parallel experiment, however, the inhibition effect of miR-218 on the luciferase reporter was totally abolished when co-transfected with a reporter vector harboring a mutated *REST* 3’-UTR (Figure 3B). These results demonstrated that miR-218 could specifically target the 3’-UTR of *REST* *in vitro*. We further observed the effect of miR-218 on controlling the expression levels of REST by real-time PCR and Western blot, respectively, in H9C2 cells, and found that upregulation of miR-218 exhibited a decreasing trend in mRNA levels of *REST* (Figure 3C) and significantly inhibited REST expression in protein levels (Figure 3D). Consistently, when reducing endogenous miR-218 by an miR-218 inhibitor, the protein level of REST could be obviously elevated (Figure 3D). In hypertrophic cardiomyocytes, we also observed that the protein level of REST was increased, but overexpression of miR-218 could significantly reduce the upregulation (Figure 3E). Meanwhile, the inhibition of miR-218 could reverse the downregulation of REST in protein levels (Figure 3F). In addition, we examined the status of REST in a hypertrophy model and found that, like the change in cardiomyocytes stimulated by ISO, REST was significantly upregulated (Figure 3G) in hearts of two-week TAC mice. All of these results strongly suggest that miR-218 involvement of hypertrophy may mediate regulation of REST to promote the pathogenesis.

## 3. Discussion

In recent years, many reports have demonstrated important roles for miRNAs in the process of hypertrophy or heart failure. For example, overexpression of miR-98 reduces cardiomyocyte size; however, knockdown of miR-98 can augment cardiac hypertrophy, respectively, under stimulation of angiotensin II [21]. miR-133 and miR-1 have been demonstrated as crucial players against hypertrophy, in which both of the miRNAs exhibit downregulation in the hypertrophy model and left ventricular tissue of patients with cardiac hypertrophy.

miR-218 has been recognized as an important player to inhibit the migration, invasion, and proliferation of tumor cells by targeting multiple cancer-associated genes and signaling pathways [22]. However, the effect of miR-218 in cardiovascular diseases is poorly understood. Previous analysis using high-though microarray had uncovered that miR-218 was really downregulated in both TAC mouse models and heart failure patients [17,18]. In the study, we further confirmed that miR-218 plays an essential role in the process of cardiac hypertrophy *in vitro* and *in vivo* as well. In addition, we also identified REST as a novel target of miR-218 to likely be involved in the pathogenesis. 

MicroRNAs generally regulate hypertrophic processes by targeting specific genes under certain conditions. So far, a variety of downstream targets of the related miRNAs have been reported. For example, miR-133 was shown to inhibit hypertrophy through targeting RhoA and Cdc42 [7]. miR-208 could induce cardiomyocyte hypertrophy by changing triiodothyronine-dependent repression of β-MHC [4]. Here, we confirm that miR-218 is an essential miRNA that participates in regulation of cardiac hypertrophy based on *in vitro* and *in vivo* data. In cardiomyocytes, we found that overexpression of miR-218 could attenuate hypertrophic extent induced by ISO, whereas reducing the endogenous level of miR-218 via transfecting the miR-218 inhibitor could exacerbate the hypertrophic response, indicating that the downregulation of miR-218 is sufficient for development of cardiomyocyte hypertrophy. Our results *in vivo* confirmed again that miR-218 was indeed downregulated in two-week TAC models with typical hypertrophy markers, which is consistent with other findings in both mice and humans. Thus, it is clear that miR-218 might play a role in the anti-hypertrophic process. The next question is how miR-218 regulates the downstream target genes to mediate the process of hypertrophy. By combining the approaches of bioinformatic analysis and luciferase assay, we identified that REST is specifically targeted by miR-218. Furthermore, the expression level of REST was increased along with the downregulation of miR-218 in hypertrophic progression, suggesting REST as a potential mediator to induce the cardiac pathogenesis.

It is well known that the process of cardiac pathological remodeling and heart failure are greatly affected by the alterations of the cardiac gene program. For instance, cardiac hypertrophy is usually accompanied by re-expression of the fetal genes such as *ANP*, *BNP* and *MYH7*, which are generally repressed in the adult heart [13,23,24]. REST has been shown to play an important role in controlling multiple fetal cardiac genes [19]. In addition, REST regulates cardiac differentiation of embryonic stem cells by regulating the *Wnt*/β-catenin signaling pathway and GATA4 [25]. In a feed forward mechanism, the *Wnt* signaling pathway is also stimulated by miR-218 through downregulating *Wnt* signal inhibitors [26]; miR-218 also shows a suppression on cell proliferation by targeting PI3K/Akt/mTOR signaling; knockdown of REST can disrupt the mTOR pathway, indicating that the miR-218-REST-mTOR pathway may be involved in the biological activity [27,28]. Indeed, upregulation of REST can promote cell proliferation [29]. On the other hand, overexpression of miR-218 inhibited NF-kappa B transcriptional activation and transcription of cyclooxygenase-2, a proliferative gene regulated by NF-κB [30]. These results strongly suggest that the downregulation of miR-218 may increase the expression of REST, which may, in turn, regulate multiple potential pathways to induce cardiac hypertrophy. Therefore, further investigation is needed to elucidate a more detailed mechanism underlying the regulation of cardiac hypertrophy by miR-218.

## 4. Material and Methods

### 4.1. Animals

All of the C57BL/6J mice were purchased from Slaccas Company (Shanghai, China). One-day-old Sprague–Dawley rats (Slaccas Company, Shanghai, China) were used to isolate cardiomyocytes. The procedures involving animals in this work were carried out in accordance with the recommendations of the guidelines for the Care and Use of Laboratory Animals from China Council on Animal Care. This study was approved by the ethical committee of the Tongji University School of Medicine (Approval No: TJLAC-014-018, Approval Date: 1 December 2014). The experiments were in accordance with the administration of affairs concerning experimental animals.

### 4.2. Pressure-Overload Hypertrophy Model

The 8–12 week old C57BL/6J mice were randomly divided into two groups: the sham-operated group and TAC-operated group. The establishment of a TAC mouse model is described in brief as following: first of all, the surgical instruments were autoclaved, and then mouse skin was prepared by shaving after inhaling isoflurane and performing trachea cannula, which linked to a respirator with the frequency of 120–140 per min. After fully exposing the surgical field, the 27 G syringe needle was inserted into the aorta and knotted surround tightly. When the syringe needle was extracted, the chest was closed by suture. The operation in the sham group was the same as that conducted in the TAC group except for the process of aortic ligation. All of the operated mice were fed in the same condition. The effect of operation was evaluated by echocardiography after 2 weeks.

### 4.3. NRCMs’ Isolation and Culture

Cardiomyocytes were isolated from 48 h newborn Sprague–Dawley rats and were digested by 0.25% trypsin. Cells were cultivated in Dulbecco’s modified Eagle’s medium (DMEM, Gibco Chemical Co., Carlsbad, CA, USA) for 24 h and transferred into culture medium with 10% FBS. Furthermore, 20 µM of miR-218 mimic, miR-218 negative control, miR-218 inhibitor and miR-218 inhibitor negative control chemical were respectively transfected into cardiomyocytes using X-tremeGENE siRNA Transfection Reagent (Roche, Mannheim, Germany) according to the protocol provided by manufacture. After 48 h, cardiomyocytes were treated with 10 µM ISO (Sigma, Saint Louis, MO, USA) for inducing hypertrophy. Moreover, H9C2 cells were cultured in the medium with 10% FBS. miR-218 mimic, miR-218 negative control, miR-218 inhibitor and miR-218 inhibitor negative control chemical with 20 µM were transfected into H9C2 cells, respectively. 

### 4.4. Determinations of Cell Surface Areas

To measure the surface area of cardiomyocytes, cells were fixed with 4% paraformaldehyde and were stained with α-actinin (1:50, Abcam, San francisco, CA, USA, ab18061). AxioVision Rel 4.4 Package (Carl Zesis, Oberkochen, Germany) was used to measure the surface area.

### 4.5. Quantitative Real-Time PCR

The total RNA was extracted from tissues and cells with TRIzol reagent (Invitrogen, Carlsbad, CA, USA). Before running quantitative PCR, 5× PrimeScript Master Mix (Takara DRR036A-1, Kusatsu, Japan) were used to reversely transcribe the RNA into cDNA. Parts of primers used in experiments are listed in Table 1. The primers of miR-218 and U6 were listed in Table 2. *GAPDH* was used as a control for normalization in the tests of the levels of hypertrophic genes. U6 was used as a control for normalization in the tests of levels of miR-218.

### 4.6. Protein Extraction and Western Blot

The total protein was extracted with RIPA solution, then separated with SDS/PAGE gel and transferred to PVDF membranes. GAPDH (1:1000, Santa Cruz, Dallas, TX, USA); NPPB (1:2000, Abcam, San Francisco, CA, USA) and REST (1:2000, Abcam, San Francisco, CA, USA) antibodies were used for immunoblot.

### 4.7. Echocardiographic Assessment of Cardiac Dimensions and Function

Mice which have undergone 2 weeks of TAC and sham operation were anesthetized with inhalational isoflurane before echocardiographic assessment. We used M-mode to execute echocardiographic assessment (Vevo770, Visual Sonics Inc., Toronto, ON, Canada) to detect the left ventricular function. We measured the left ventricular internal diameter at diastole and systole (LVIDd and LVIDs, respectively) in at least three beats from each projection. The fractional shortening (FS) of the left ventricle was defined as 100% × (1-LVIDs/LVIDd). We used a two-dimensional short-axis view of the left ventricle and M-mode tracings to measure the anterior and posterior wall thickness of left ventricular (LVAWD, LVPWD) at end-diastole and papillary muscle level. Left ventricle ejection fraction (LVEF) was obtained using the Simpson approach.

### 4.8. Luciferase Assay

After obtaining wild-type and mutant *REST* 3’-UTR sequences (NCBI Reference Sequence: NM_031788), we respectively engineered these inserts into the PMIR vector that contains fragments of luciferase (AM5795, Invitrogen). In addition, 2 µM of each miR-218 mimic or miR-218 inhibitor was cotransfected into H9C2 cells with PMIR-*REST* or PMIR-*REST*mut (20 μg/mL) (Thermo Scientific DharmaFECT^®^ Duo Transfection Reagent, Carlsbad, CA, USA). At 48 h after transfection, we lysed the cells and measured the luciferase activity by luciferase reporter assay system (Beyotime Biotechnology, Shanghai, China). All of the experiments were performed in triplicate. The H9C2 cell line was provided by the Min Yu research group from the Life Science school in Fudan University.

### 4.9. Statistical Analysis

Data are displayed as the means ± SEM in three independent experiments. The Student’s *t*-test was used to assess the differences of two groups. The threshold value of statistical significance (*p*) was set at 0.05 or below.

## 5. Conclusions

In conclusion, our work shows that miR-218 is downregulated during cardiac hypertrophy induced by TAC in mice, and overexpression of miR-218 can attenuate hypertrophy in cardiomyocytes induced by ISO. Moreover, miR-218 negatively regulates expression of REST *in vitro*. Based on our collective findings, we suggest that our results provide an important foundation for further exploring the signals that induce cardiac hypertrophy to provide a point of intersection for potential future therapies.

## Figures and Tables

**Table 1:** Real-time PCR primer sequences.

Gene	Forward Primer (5′→3′)	Reverse Primer (5′→3′)
*Rat-ANP*	GGAAGCTGTTGCAGCCTA	GCCCTGAGCGAGCAGACCGA
*Rat-BNP*	CTTTTCCTTAATCTGTCGCCG	GTCTCTGAGCCATTTCCTCTG
*Rat-MYH7*	ACAACCCCTACGATTATGCG	CGCCTGTCAGCTTGTAAATG
*Rat-REST*	ACTTTGTCCTTACTCAAGTTCTCAG	ATGGCGGGTTACTTCATGTT
*Rat-GAPDH*	AACGACCCCTTCATTGACCTC	CCTTGACTGTGCCGTTGAACT
*Mouse-ANP*	TCCTCGTCTTGGCCTTTTG	CTCATCTTCTACCGGCATCTTC
*Mouse-BNP*	GCACAAGATAGACCGGATCG	CCCAGGCAGAGTCAGAAAC
*Mouse-MYH7*	CCATCTCTGACAACGCCTATC	GGATGACCCTCTTAGTGTTGAC
*Mouse-REST*	AACGAGAAGATGGAGAATGA	CACTGAGACACTGCTACC
*Mouse-GAPDH*	TTTGCAGTGGCAAAGTGGAGATT	CCCATTTGATGTTAGTGGGGTCTCG

**Table 2:** Primers of miR-218 and U6.

Use	Sequences (5′-3′)
mmu-miR-218	RT: GTCGTATCCAGTGCAGGGTCCGAGGTATTCGCACTGGATACGACACATGG
	Forward: TGTGCTTGATCTAACCATGTGTC
	Reverse: CCAGTGCAGGGTCCGAGGTA
U6	RT: AACGCTTCACGAATTTGCGT
	Forward: CTCGCTTCGGCAGCACA
	Reverse: AACGCTTCACGAATTTGCGT

## Abbreviations

miRNA	microRNA
ISO	isoprenaline
REST	RE1-silencing transcription factor
TAC	transverse aortic constriction
3’-UTR	3’-untranslated region
FBS	fetal calf serum
ANP	atrial natriuretic peptide
BNP	brain natriuretic peptide
MYH7	b-myosin heavy chain
LVIDd	left ventricular internal dimensions at diastole
LVIDs	left ventricular internal dimensions at systole
FS	fractional shortening
LVAWD	left ventricular anterior wall thickness
LVPWD	left ventricular posterior wall thickness
LVEF	left ventricle ejection fraction
lncRNA	long non-coding RNA
